# Neuroprotective Effects of N-Acetyl-Cysteine and Acetyl-L-Carnitine after Spinal Cord Injury in Adult Rats

**DOI:** 10.1371/journal.pone.0041086

**Published:** 2012-07-17

**Authors:** Amar Karalija, Liudmila N. Novikova, Paul J. Kingham, Mikael Wiberg, Lev N. Novikov

**Affiliations:** 1 Department of Integrative Medical Biology, Section of Anatomy, Umeå University, Umeå, Sweden; 2 Department of Surgical and Perioperative Science, Section of Hand and Plastic Surgery, Umeå University, Umeå, Sweden; The Chinese University of Hong Kong, Hong Kong

## Abstract

Following the initial acute stage of spinal cord injury, a cascade of cellular and inflammatory responses will lead to progressive secondary damage of the nerve tissue surrounding the primary injury site. The degeneration is manifested by loss of neurons and glial cells, demyelination and cyst formation. Injury to the mammalian spinal cord results in nearly complete failure of the severed axons to regenerate. We have previously demonstrated that the antioxidants N-acetyl-cysteine (NAC) and acetyl-L-carnitine (ALC) can attenuate retrograde neuronal degeneration after peripheral nerve and ventral root injury. The present study evaluates the effects of NAC and ALC on neuronal survival, axonal sprouting and glial cell reactions after spinal cord injury in adult rats. Tibial motoneurons in the spinal cord were pre-labeled with fluorescent tracer Fast Blue one week before lumbar L5 hemisection. Continuous intrathecal infusion of NAC (2.4 mg/day) or ALC (0.9 mg/day) was initiated immediately after spinal injury using Alzet 2002 osmotic minipumps. Neuroprotective effects of treatment were assessed by counting surviving motoneurons and by using quantitative immunohistochemistry and Western blotting for neuronal and glial cell markers 4 weeks after hemisection. Spinal cord injury induced significant loss of tibial motoneurons in L4–L6 segments. Neuronal degeneration was associated with decreased immunostaining for microtubular-associated protein-2 (MAP2) in dendritic branches, synaptophysin in presynaptic boutons and neurofilaments in nerve fibers. Immunostaining for the astroglial marker GFAP and microglial marker OX42 was increased. Treatment with NAC and ALC rescued approximately half of the motoneurons destined to die. In addition, antioxidants restored MAP2 and synaptophysin immunoreactivity. However, the perineuronal synaptophysin labeling was not recovered. Although both treatments promoted axonal sprouting, there was no effect on reactive astrocytes. In contrast, the microglial reaction was significantly attenuated. The results indicate a therapeutic potential for NAC and ALC in the early treatment of traumatic spinal cord injury.

## Introduction

Traumatic spinal cord injury affects several thousand people worldwide every year and the main reasons are motor vehicle accidents and falls [1]. The mortality after spinal cord injury is nearly 50% and about 15–20% of the patients surviving the initial trauma will die later in hospital [2]. The patients are often young and left with paralysis, spasticity and loss of sphincter control. The accumulated number of these patients, not being able to work and live a normal life, is high and many of them are in need of life-long rehabilitation and therapy [2].

In the acute phase of spinal cord injury, the initial mechanical trauma damages the blood-brain barrier and neuronal tracts resulting in interruptions of blood flow, edema, hemorrhage and retrograde reaction in axotomized neurons. Pro-inflammatory cytokines, glutamate and reactive oxygen species such as peroxynitrite and superoxide radicals are produced in the injured spinal cord tissue leading to axonal swelling, myelin breakdown, inflammation and mitochondrial dysfunction followed by apoptotic death of neurons and glial cells [3]–[6]. Free radicals and nitric oxide generated by microglia also stimulate astrocytes to secrete growth-inhibitory proteoglycans forming the astroglial scar [7] and effectively blocking axonal regeneration across the lesion site [8], [9].

At present, there are no proven pharmacological therapies for human spinal cord injury that provide neuroprotection and stimulate axonal growth [5], [10], [11]. However, since oxidative damage is a known mechanism for secondary degeneration in acute spinal cord injury, administration of antioxidants during the first hours or days after insult has been shown to stabilize mitochondrial bioenergetics, spare nerve tissue and, in some cases, result in modest neurological recovery [5], [11]–[15].

In our previous investigations we have demonstrated that prolonged 4–8 weeks treatment with the antioxidantsN-acetyl-cysteine (NAC) or acetyl-L-carnitine (ALC) can significantly reduce early retrograde death of spinal motoneurons after ventral root avulsion and delayed the degeneration of sensory DRG neurons after sciatic nerve injury [16]–[19].

NAC, a thiol-containing compound, has a broad range of actions which includes antioxidant activity, enhancement of intracellular glutathione levels and anti-inflammatory properties [20], [21]. ALC is a small water-soluble peptide located in the mitochondria. It contains a carnitine moiety which is important for the oxidation of fatty acids and an acetyl moiety which is involved in maintenance of acetyl-CoA levels and can promote the production of the antioxidant glutathione [6], [11], [22]. Both antioxidants can cross the blood-brain barrier. NAC has been used in clinical practice for many years mainly as a mucolytic agent and for paracetamol intoxication [23]. ALC has been tested clinically for age-related neurological disorders and diabetic neuropathy [24], [25].

The present study investigates the effects of continuous intrathecal infusions of NAC and ALC on degeneration of spinal motoneurons, axonal sprouting and reaction of glial cells after spinal cord hemisection in adult rats.

## Results

### Effects of Antioxidants on Neuronal Survival

**Figure 1:**
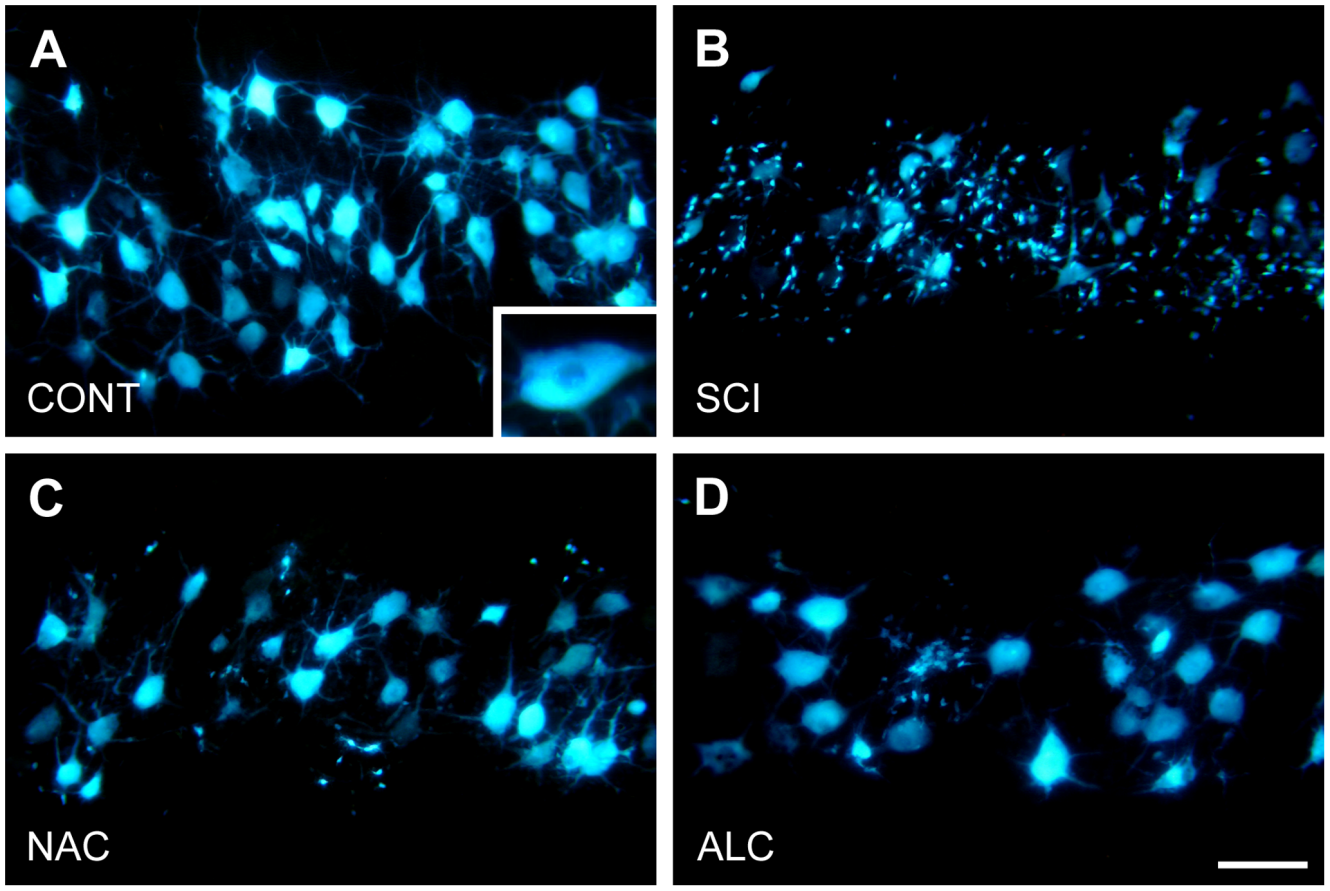
Retrograde labeling of tibial motoneurons. Horizontal sections through the L4–L5 spinal cord segments showing Fast Blue-labeled tibial motoneurons of a control animal (A; CONT), at 4 weeks after spinal cord injury (SCI) and following treatment with N-acetyl cysteine (NAC) or acetyl-L-carnitine (ALC). Insert in (A) displays pattern of Fast Blue labeling in the cell body. Scale bar, 100 µm.

**Figure 2:**
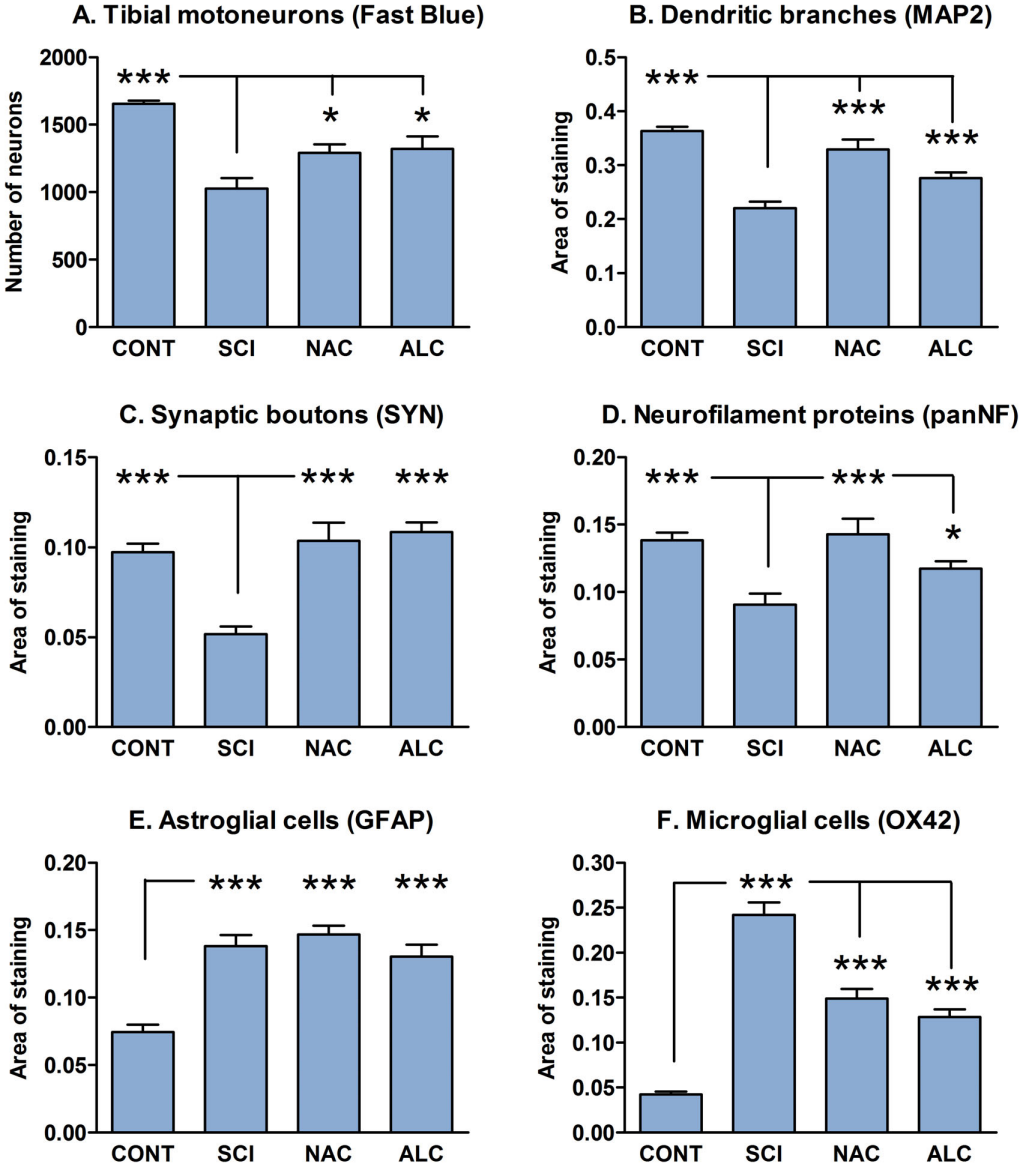
Quantification of neuronal degeneration and reaction of glial cells. Histogram showing survival of Fast Blue-labeled tibial motoneurons (A) and relative tissue area occupied by MAP2-positive dendritic branches (B), synaptophysin-positive synaptic boutons (C), neurofilament-positive nerve fibers (D), GFAP-positive astrocytes (E) and OX42-positive microglial cells (F) in the L5 ventral horn of uninjured control rats (CONT), at 4 weeks after spinal cord injury (SCI) and following treatment with N-acetyl cysteine (NAC) or acetyl-L-carnitine (ALC). Error bars show S.E.M. P<0.05 is indicated by*, P<0.01 is indicated by** and P<0.001 is indicated by*** (SCI vs. CONT, NAC, ALC in A–D and F; CONT vs. SCI, NAC, ALC in E).

To evaluate neuroprotective effects of NAC and ALC on neuronal survival, we pre-labeled tibial motoneurons with fluorescent dye Fast Blue. In accordance with previous observations, all Fast Blue-labeled motoneurons were found in the L4–L6 spinal cord segments [26], [27], where they formed a 5.3±0.3 mm long cell column. In control uninjured animals, at 1 week after Fast Blue application to the cut peripheral nerve, the tibial motoneuron pool contained 1656±23 labeled cell bodies with clearly visible primary and secondary dendrites (Fig. 1A). Spinal cord hemisection at L5 lumbar level induced significant cell death and only 62% of labeled motoneurons remained after 4 weeks (Fig. 1B and Fig. 2A). Counting of labeled cells within 2 mm distance rostral and caudal to the lesion site revealed an increase in the rate of degeneration among rostral motoneurons (rostral: 233±33 motoneurons; caudal: 358±32 motoneurons; mean±SEM; P<0.05). In addition, surviving motoneurons lost Fast Blue labeling in their dendritic branches and were surrounded by numerous small Fast Blue-positive cells most likely representing activated microglial cells (Fig. 1B). NAC treatment resulted in 78% survival (Fig. 2A) of the tibial motoneurons. Labeling of primary dendrites was partially preserved and the number of Fast Blue-labeled microglia-like cells was decreased (Fig. 1C). ALC treatment had similar neuroprotective effect on tibial motoneurons (80% survival, Fig. 1D and Fig. 2A), however, preservation of Fast Blue labeling in primary and secondary dendrites was less evident.

### Effects of Antioxidants on Dendrites and Synaptic Boutons

**Figure 3:**
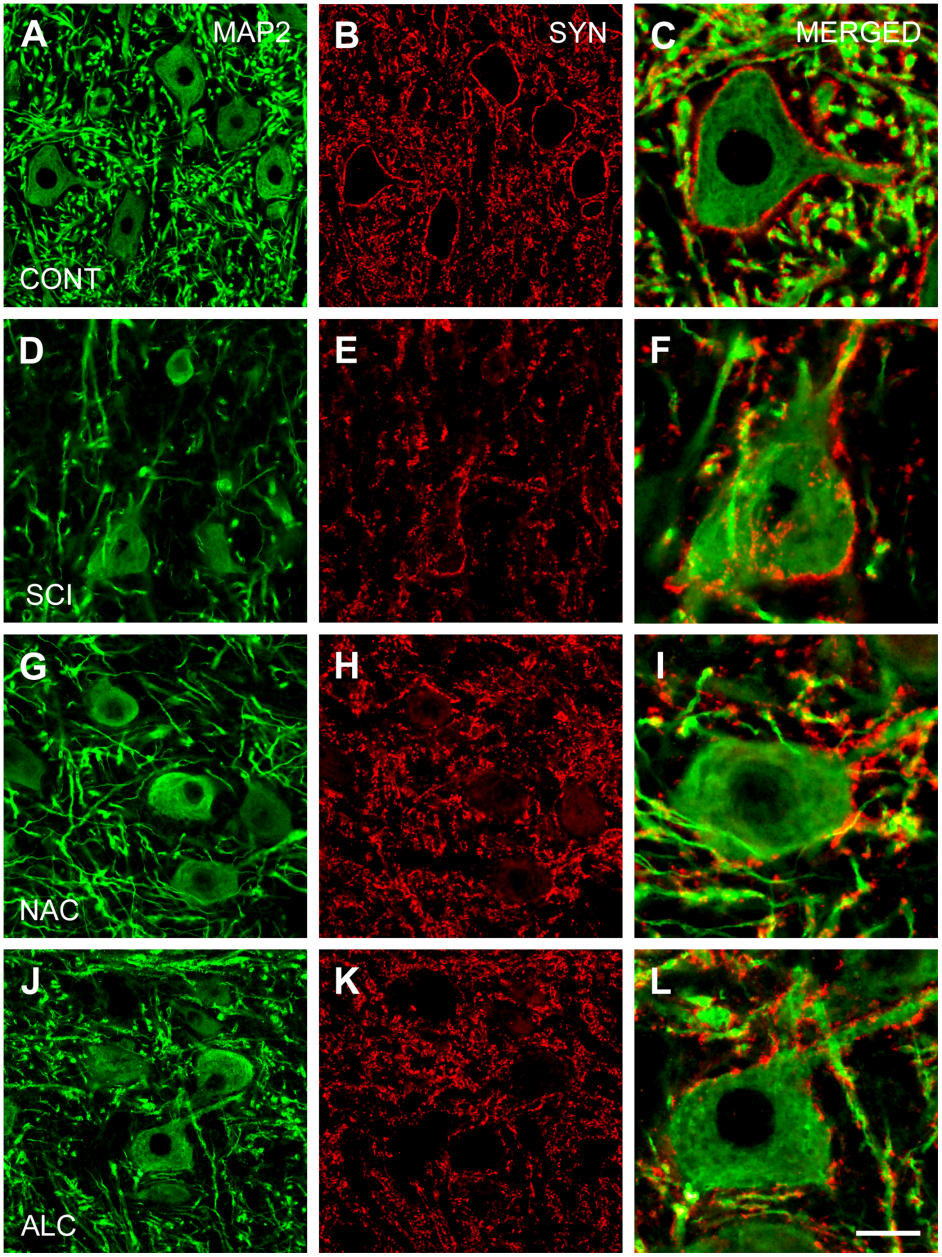
Expression of microtubule-associated protein-2 and synaptophysin. Horizontal sections through the ventral horn of L4–L5 segments showing immunostaining for microtubular-associated protein-2 (MAP2; dendritic branches, left column) and synaptophysin (SYN; synaptic boutons, middle column) of a control animal (A–C; CONT), at 4 weeks after spinal cord injury (D–F; SCI) and following treatment with N-acetyl cysteine (G–I; NAC) or acetyl-L-carnitine (J–L; ALC). Note that synaptic boutons around motoneuron cell bodies are not recovered after NAC or ALC treatment (right column). Scale bar, 50 µm (left and middle columns) and 20 µm (right column).

Quantification of preparations immunostained for MAP2 and synaptophysin in the L4–L5 spinal segments of control animals revealed that about 36.3%±0.9% and 9.7%±0.4% of ventral horn neuropil was occupied by dendritic branches and synaptic boutons, respectively (Fig. 2B,C and Fig. 3 A–C). Distinct pre-synaptic synaptophysin labeling was observed around neuronal cell bodies and proximal dendrites (Fig. 3C), while the ventral horn neuropil displayed more diffuse staining pattern. Spinal cord injury significantly decreased MAP2 and synaptophysin immunostaining by 39% and 47%, respectively (P<0.05; Fig. 2B,C and Fig. 3D,E). In addition, a reduction of synaptophysin labeling around motoneuron cell bodies and proximal dendrites was found (Fig. 3F). Treatment with NAC restored MAP2 and synaptophysin immunoreactivity to 89% and 107% of control values, respectively (P<0.05; Fig. 2B,C and Fig. 3G,H). ALC demonstrated similar effects on dendritic branches and synaptic boutons with return to 76% and 111% of control MAP2 and synaptophysin staining, respectively (P<0.05; Fig. 2B,C and Fig. 3J,K). However, both antioxidants failed to recover typical pattern of synaptophysin labeling around cell bodies (Fig. 3I,L).

### Effects of Antioxidants on Axonal Sprouting and Glial Reaction

**Figure 4:**
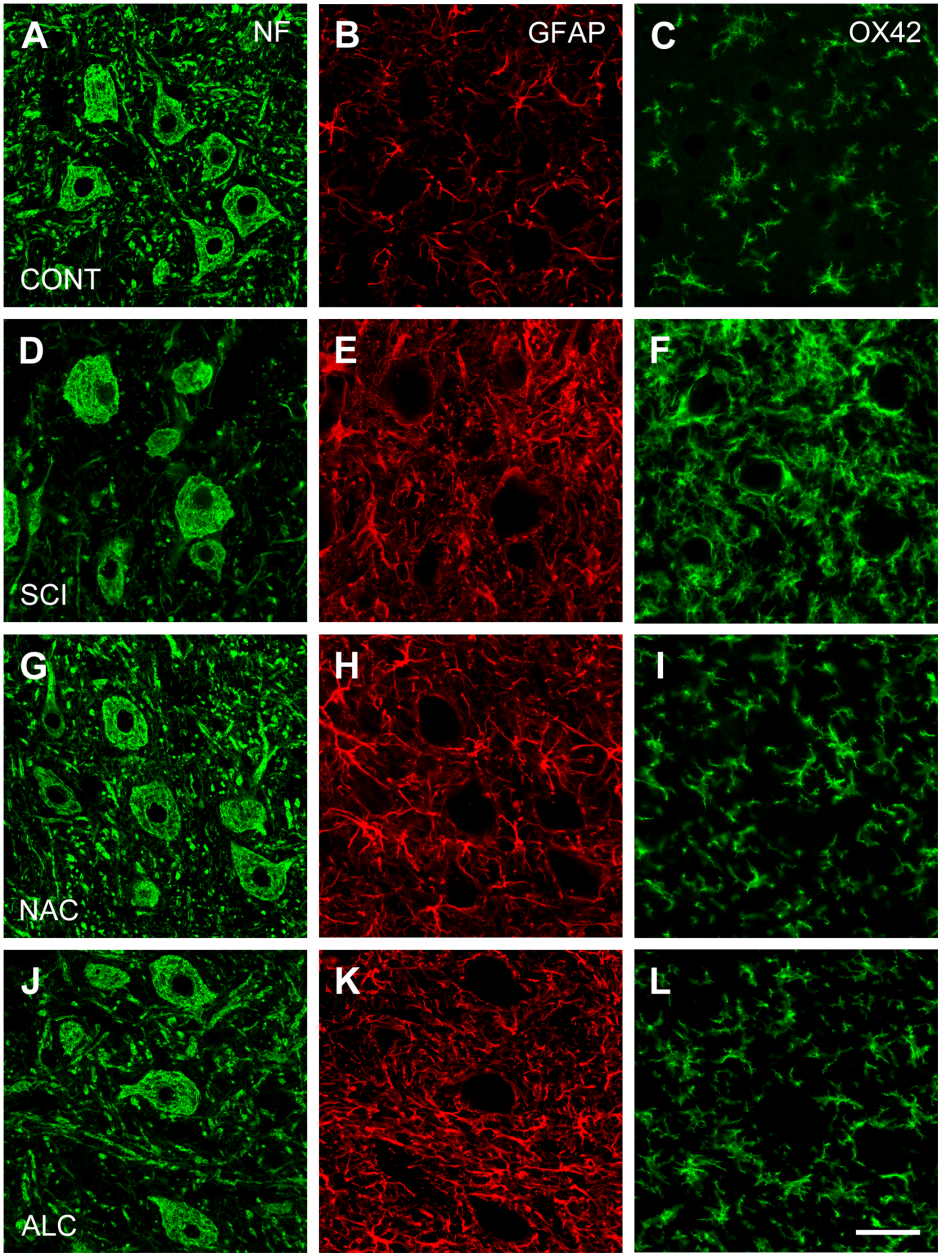
Expression of neurofilament proteins, glial fibrillary acidic protein and microglial complement C3bi receptor. Horizontal sections through the ventral horn of L4–L5 segments showing immunostaining for neurofilaments (NF; nerve fibers; left column), glial fibrillary acidic protein (GFAP; astrocytes, middle column) and microglial complement C3bi receptor (OX42; microglial cells; right column) of a control animals (A–C; CONT), at 4 weeks after spinal cord injury (D–F; SCI) and following treatment with N-acetyl cysteine (G–I; NAC) or acetyl-L-carnitine (J–L; ALC). Note that NAC and ALC did not change GFAP immunoreactivity but significantly decreased OX42 immunostaining. Scale bar, 50 µm.

In control rats, the area of the ventral horn occupied by pan-neurofilament-positive axons, GFAP-positive astroglial cells and OX42-positive microglial cells was 13.8%±0.6%, 7.4%±0.5% and 4.2%±0.3%, respectively (Fig. 2D,E,F and Fig. 4A,B,C). Spinal cord hemisection decreased pan-neurofilament immunostaining by 34% (P<0.05) but significantly increased GFAP and OX42 immunoreactivity in the L4–L5 ventral horn neuropil rostral to the injury site (Fig. 2D,E,F and Fig. 4D,E,F; P<0.05; 187% and 576%, respectively). Treatment with NAC restored pan-neurofilament immunostaining to the normal values (104%, P<0.05; Fig. 2D and Fig. 4G), whereas ALC was less effective (86% recovery; P<0.05; Fig. 2D and Fig. 4J). GFAP immunostaining was not affected by antioxidant treatment (Fig. 2E and Fig. 4H,K). However, both NAC and ALC significantly attenuated OX42 immunoreactivity in the ventral horn (P<0.05; Fig. 2F and Fig. 4 I,L).

**Figure 5:**
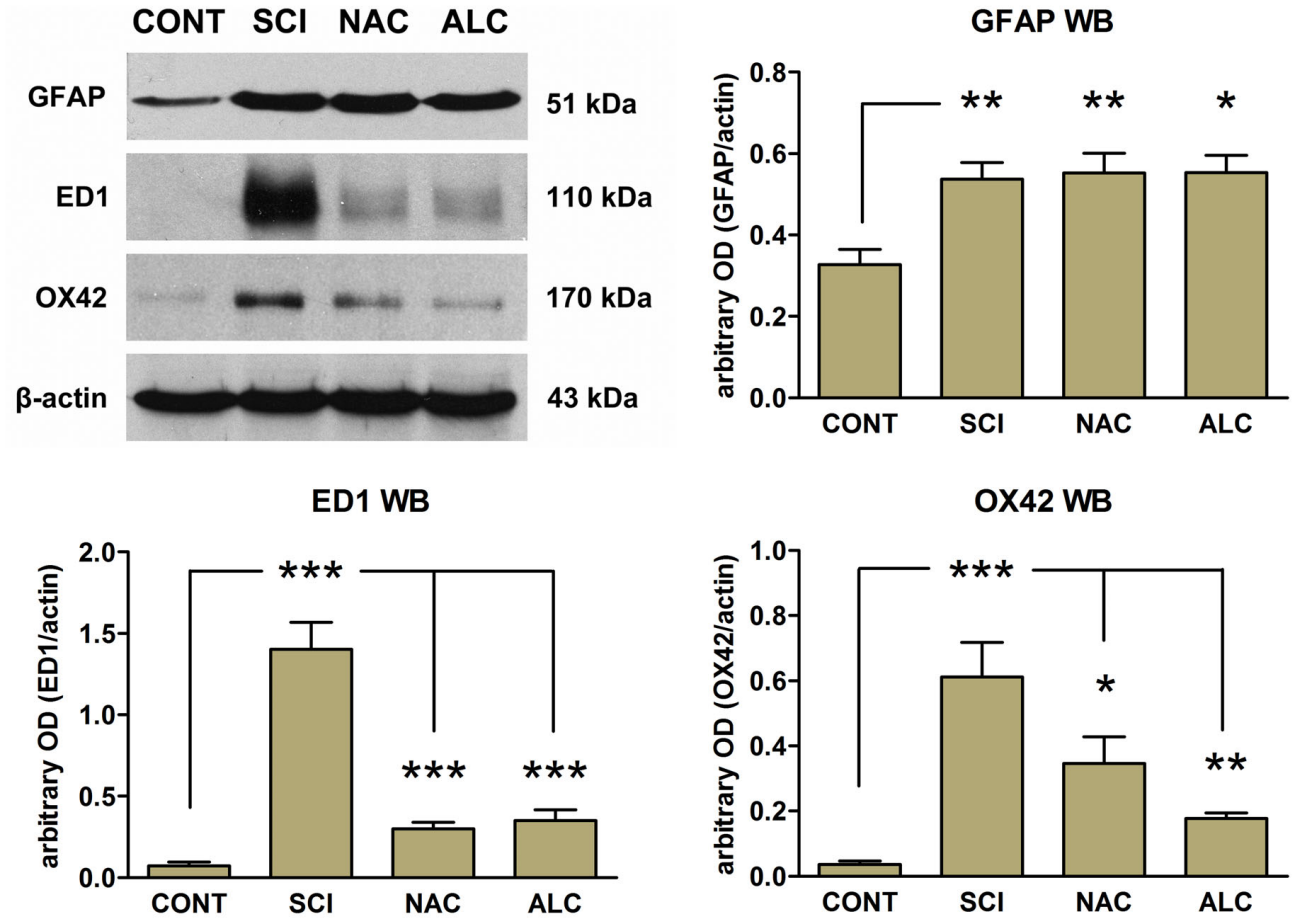
Representative Western blots for GFAP, ED1 and OX42 expression in L4–L5 spinal cord segments. Protein was prepared from healthy uninjured control rats (CONT) or rats subjected to L5 spinal cord injury in the absence (SCI) or presence of N-acetyl cysteine (NAC) or acetyl-L-carnitine (ALC) treatment. Histograms show the mean optical density (OD) for each band expressed relative to the corresponding β-actin levels for each sample, for a total of 4 animals per group. Error bars show S.E.M. P<0.05 is indicated by*, P<0.01 is indicated by**and P<0.001 is indicated by***(CONT vs. SCI, NAC, ALC for GFAP; SCI vs, CONT, NAC, ALC for ED1 and OX42).

The effects of NAC and ALC on astroglial and microglial cells were also evaluated using Western blot analysis of the L4–L5 hemicords rostral to the lesion site (Fig. 5). Similar to immunofluorescence studies, spinal cord injury resulted in up-regulation of GFAP (P<0.01; Fig. 5) but these protein levels were not altered by treatment with NAC or ALC (P>0.05; Fig. 5). In contrast, expression of ED1 (a marker of activated microglia/macrophages) and OX42 were barely detected in control tissue from uninjured control animals and were up-regulated following spinal cord injury (P<0.001; Fig. 5). Treatment of the animals with NAC or ALC significantly down-regulated the expression levels of ED1 (P<0.001) and OX42 (P<0.05 for NAC and P<0.01 for ALC).

## Discussion

This study is the first demonstration of the neuroprotective efficacy of NAC and ALC treatments to reduce the degeneration of spinal motoneurons, and restore the density of dendritic branches and axonal terminals in the ventral horn of the hemisected spinal cord. Although the antioxidants tested do not affect astrocytes, they significantly attenuate the reaction of activated microglial cells.

It is well known that spinal cord injury results in cell death among spinal neurons and glial cells [28]–[30]. As early as two weeks after low-thoracic spinal injury, significant loss of remote long and short descending propriospinal tract neurons has been demonstrated [31]. In addition, spinal cord injury leads to the delayed retrograde reaction in descending motor and ascending sensory spinal tract neurons [32].

While our study confirmed previous observations that spinal cord injury induces degeneration of spinal motoneurons with loss of dendritic branches, axonal terminals and presynaptic boutons [27], [30], [33]–[35], it also demonstrated that the degenerative processes rostral and caudal to the injury epicenter progressed asymmetrically with significantly less motoneurons remaining above the lesion site. The latter could be due to the oblique caudal course of the motor axons in the ventral white matter before leaving the spinal cord [36]. Therefore, during spinal cord hemisection, these axons will undergo “intramedullary” lesion, a very proximal type of axonal injury leading to rapid retrograde death of corresponding motoneurons [37], [38].

The irreversible loss of central neurons is an important contributing factor to the subsequent permanent deficit of neurological function and therefore is a potential target for future neuroprotective treatment strategies. Different experimental therapeutic approaches have been tested in order to minimize the progressive cell loss after spinal cord injury. It has been shown that neurotrophic factors can prevent neuronal degeneration and support axonal sprouting [27], [32]. Recently, it has also been demonstrated that transplantation of cultured stem cells and olfactory ensheathing cells could have significant neuroprotective effect after traumatic spinal cord injury [39]–[41].

Following injury to the central nervous system, mitochondrial dysfunction and generation of reactive oxygen species could lead to activation of neuronal and glial cell apoptosis [6], [42], [43]. The time course of oxidative stress indicates that antioxidant treatment must be initiated within the first hours after spinal cord injury and maintained for at least 1–2 weeks to attenuate long-term effects of protein nitrative damage and lipid peroxidation [5]. The antioxidants ALC and NAC are well known for their neuroprotective effects and they can both enhance neuronal biosynthesis of glutathione which is the principal renewable free radical scavenger within neurons [11], [20], [22], [44]. It has also been shown that ALC can be used as an alternative substrate in mitochondrial respiration in the absence of pyruvate [12]. In rats with spinal cord contusion, intraperitoneal administration of ALC during the first 6 hours post-injury maintains mitochondrial respiration and enzyme activities. Moreover continuous treatment with ALC for 1 week spares spinal cord gray matter [12]. Administration of ALC and NAC during the first 24 hours has also been shown to reduce the volume of lesion after traumatic brain injury [45], [46]. Both antioxidants have in addition been tested as early neuroprotective treatments after ischemic spinal cord injury and provide significant reduction of motor dysfunction [47]–[49]. In the present study, we administered either NAC or ALC intrathecally for 4 weeks using osmotic minipumps and found marked neuroprotective effect on lesioned spinal motoneurons with almost complete restoration of density of dendritic branches and axonal terminals in the ventral horn. However, in contrast to the neurotrophic factor BDNF [50], treatment with antioxidants did not restore the typical pattern of perineuronal synaptophysin immunoreactivity formed by axosomatic synaptic boutons.

In addition to direct reductant and free radical scavenging effects, NAC could also signal through the neurotrophic factor-activated Ras-ERK pathway and stress-activated JNK pathways [51]–[53]. ALC has been shown to enhance the response of PC12 cells to NGF by stimulating the synthesis of NGF receptors [54]–[56]. These data suggest that NAC and ALC can share the capacity to maintain neuronal survival with neurotrophic factors, which has been demonstrated in experiments with peripheral nerve injury. Retrograde neuronal death after nerve injury is largely due to diminished neurotrophic signaling from remaining Schwann cells and target structures [57]. In dorsal root ganglia (DRG), most of the sensory neurons survive the initial 2–4 week post-injury but only 50% of neurons remain after 13–16 weeks [58], [59]. However, 8 weeks of continuous treatment with either NAC or ALC significantly reduces this retrograde degeneration of sensory neurons [16], [17]. Moreover, effects of NAC treatment include preservation of the mitochondrial architecture in sensory cells and reduction of the number of TUNEL-positive neurons and glial cells [60]. The neuroprotective effect of NAC has been associated with considerable down-regulation of Bax and caspase-3 mRNA and up-regulation of Bcl-2, peripherin and activating transcription factor 3 [61], [62]. Although sciatic nerve injury in adult rodents does not induce significant cell death among spinal motoneurons, facial nerve axotomy in newborns and ventral root avulsion in adults causes massive cell loss which could be attenuated by ALC and NAC [18], [63]. In another long-term study, NAC was administered for 12 months in the drinking water to the EAAC1(−/−) mice (model of Parkinson’s disease) and was found to reduce substantially age-dependent loss of dopaminergic neurons in the substantia nigra [64].

Spinal cord injury is invariably associated with reactive astrogliosis and activation of microglia and macrophages [7], [65]. In our study, spinal cord hemisection caused an almost two-fold increase in GFAP-immunoreactivity in astrocytes and six-fold increase in OX42-immunoreactivity in microglial cells. However, despite the marked survival effect on spinal motoneurons, ALC and NAC did not affect GFAP levels. In contrast, both antioxidants reduced the microglial reaction. The mechanisms underlying the lack of effects of NAC or ALC on reactive astrocytes after spinal cord injury are not clear. They could be attributed to the well known pro-survival effects of these antioxidants on cultured astrocytes following oxidative stress [66]–[69]. The effect of ALC and NAC on microglia and macrophages after spinal cord injury could be secondary to their survival effects on neurons and glial cells. Restoration of the dendritic branches, axonal arborizations and presynaptic boutons could also contribute to this effect.

Studies in vitro [70] and in vivo [71] demonstrate that NAC can suppress expression of several important neuroinflammatory molecules including matrix metalloproteinases, TNF-alpha, interleukin 1beta and inducible nitric oxide synthase in lipopolysaccharide-stimulated microglial cells and in a rat model of experimental stroke. Moreover, NAC has been found to reduce expression of ED1 in activated microglial cells and macrophages [71]. ALC could also reduce inflammatory reactions in the brain by lowering circulating levels of TNF-alpha and interleukins [72]. Recently, a new role of mitochondria in inflammatory response has been identified [73]. Reactive oxygen species produced in mitochondria have been shown to activate the NLRP3 inflammasome and trigger innate immune defences via pro-inflammatory cytokines. Therefore, stabilization of mitochondria following antioxidant treatment could attenuate inflammatory processes and decrease the reaction of microglia and macrophages in the injured spinal cord.

In summary our results show that continuous intrathecal infusion of the antioxidants, N-acetyl-cysteine and acetyl-L-carnitine, reduces neuronal degeneration in the ventral horn and attenuates the microglial reaction and inflammation after spinal cord injury in adult rats. Since both antioxidants have been used in clinical practice for many years, they represent a promising and safe neuroprotective strategy for human spinal cord injury.

## Materials and Methods

### Experimental Animals and Ethics Statement

The experiments were performed on adult (10–12 weeks, n = 56, Table 1) female Sprague-Dawley rats (Taconic Europe A/S, Denmark). The animal care and experimental procedures were carried out in accordance with the European Communities Council Directive (86/609/EEC) and were also approved by the Northern Swedish Committee for Ethics in Animal Experiments (Permit Number: A127-10). All surgical procedures were performed under general anesthesia using a mixture of ketamine (Ketalar®, Parke-Davis; 100 mg/kg i.v.) and xylazine (Rompun®, Bayer; 10 mg/kg i.v.). After surgery the rats were given the analgesic Finadyne (Schering-Plough, Denmark; 2.5 mg/kg, i.m.), normal saline (2 ml s.c.) and benzylpenicillin (Boehringer Ingelheim; 60 mg i.m.).

**Table 1:** Experimental groups and numbers of animals.

Experimental groups	Abbreviations used in figures	Retrograde labelingwith Fast Blue	Immuno histochemistry	Western blotting
Normal animals	CONT	6	4	4
Spinal cord injury	SCI	6	5	4
SCI + N-acetyl-cysteine	NAC	5	4	4
SCI + Acetyl-L-carnitine	ALC	6	4	4

### Retrograde Labeling of Spinal Motoneurons

In the experiments studying neuronal survival (n = 23), tibial motoneurons in the L4–L6 spinal segments [26], [27] were labeled with non-toxic retrograde fluorescent tracer Fast Blue (FB, EMS-Chemie GmbH, Germany) one week before L5 spinal cord injury. The tibial nerve was transected in the popliteal fossa and the proximal stump was introduced into a small polyethylene tube containing 2% aqueous solution of the dye [74]. The tube was sealed with a mixture of silicone grease and Vaseline to prevent leakage and the tracer was left in contact with the cut nerve for 2 hours. The tube was then removed, the nerve rinsed in saline and the muscle and skin sutured.

### Spinal Cord Injury

After lumbar laminectomy, the L5 spinal cord segment was identified and vertically penetrated with a 23 G needle in the dorsal root entry zone. After introducing one blade of a pair of Vannas spring scissors into the stab wound, the other blade was used to transect the lateral funiculus and adjacent gray matter from the lateral side. Special care was taken to avoid damage to dorsal and ventral roots. Dura mater was covered with stretched parafilm and Spongostan®, and then muscles and skin were closed in layers. The rats were randomly divided into three experimental groups (Table 1): (i) spinal cord injury without treatment (SCI, n = 15), (ii) SCI followed by treatment with N-acetyl-cysteine (NAC, n = 13) and (iii) SCI followed by treatment with acetyl-L-carnitine (ALC, n = 14). The animals tolerance to L5 SCI was good and only one rat was lost in the group “SCI + NAC treatment”. Eight healthy uninjured rats (immunohistochemistry and Western blotting) and 6 rats at 1 week after Fast Blue labeling served as baseline controls.

### Treatment with N-acetyl-cysteine and Acetyl-L-carnitine

Immediately after the injury, an Alzet 2002 osmotic minipump (Alza Corp., Palo Alto, CA) filled with clinically available solution of L-stereoisomer of N-acetyl-cysteine (200 mg/ml; BioPhausia) or O-acetyl-L-carnitine hydrochloride (75 mg/ml in normal saline; Sigma-Aldrich) was implanted subcutaneously in the neck. After partial L6 laminectomy, a subcutaneous polyethylene catheter (Intermedic, PE-60) was inserted into the lower lumbar subarachnoid space [17], [75]. The catheter tip was placed at the level of L3–L4 dorsal root ganglia, the tube was fixed to the S1 vertebral bone by Histoacryl® glue, and secured to the back muscles by several sutures. The implantation site was covered with Spongostan® and the wound was closed in layers. The pump infusion speed corresponded to 2.4 mg/day of NAC and 0.9 mg/day of ALC. The doses of NAC and ALC were based on our previous observations [16]–[18]. After 14 days of continuous infusion, the emptied pump was replaced by a second pump containing the same solution. In our previous studies we have found that intrathecal infusion of vehicle solutions (PBS or normal saline) do not affect survival of spinal motoneurons following injury to the spinal cord and ventral roots [18], [38], [76]. Animals were sacrificed 4 weeks after spinal cord injury and treatment.

### Tissue Processing

At the end of experiment, the animals were deeply anaesthetized with an intraperitoneal overdose of sodium pentobarbital (240 mg/kg, Apoteksbolaget, Sweden). For Western blotting, L4–L5 spinal cord segments rostral to the injury site were divided into two halves in sagittal plane and immediately frozen in liquid nitrogen. All other animals were transcardially perfused with Tyrode’s solution followed by 4% paraformaldehyde in 0.1 M phosphate buffer (pH 7.4). Spinal cord segments L4–L6 were removed, post-fixed in the same solution, cryoprotected in 10% and 20% sucrose for 2–3 days and frozen in liquid isopentane. For counts of Fast Blue-labeled motoneurons, 50-µm-thick serial horizontal sections were cut on a vibratome (Leica Instruments), mounted on gelatin-coated glass slides, air dried, briefly immersed in xylene and coverslipped in DPX (Kebo Lab AB, Sweden). For immunohistochemistry, 16-µm-thick serial horizontal sections were cut on a cryomicrotome (Leica Instruments), thaw-mounted in pairs onto SuperFrost®Plus slides, dried overnight at room temperature and stored at −85°C before processing.

### Counts of Fast Blue-labeled Tibial Motoneurons

Retrogradely labeled tibial motoneurons were identified using a Leitz Aristoplan fluorescent microscope fitted with UV filter block A (excitation wavelength range between 340 and 380 nanometers and emission wavelength is 430 nanometers). Fast Blue is typically found in the cell body and proximal dendrites of the labeled neurons while the nuclei always remain unstained (see insertion in Figure 1A). In each section, the number of motoneurons with nuclei was manually counted at 250x final magnification and the total number of labeled profiles was calculated. In cases of very strong cytoplasmic labeling masking unstained nuclei, sections were left to fade for an additional 2–3 minutes and then examined again. To assess possible differences in reaction of motoneurons above and below the lesion site, labeled profiles were counted within 2 mm distance rostral and caudal to the middle of hemisection cavity. The total number of profiles was not corrected for split nuclei since the nuclear diameters were small in comparison with the section thickness used [75], [77], [78]. We have previously demonstrated that the accuracy of this counting technique in estimation of retrograde neuronal cell death is similar to that obtained with the physical disector method [79] and counts of neurons reconstructed from serial sections [38].

### Immunohistochemistry

Sections were processed for immunohistochemical demonstration of neuronal and glial markers. After blocking with normal serum, the following primary antibodies were used: mouse anti-microtubule-associated protein-2 (MAP2; 1∶100, Chemicon), rabbit anti-synaptophysin (SYN; 1∶500, Dako), rabbit anti-glial fibrillary acidic protein (GFAP; 1∶500; Dako), monoclonal antibodies reacting with C3bi complement receptors (OX42; 1∶250, Serotec) and a cocktail of monoclonal antibodies reacting with 68 kDa, 160 kDa and 200 kDa neurofilament proteins (NF; 1∶200; Zymed Laboratories). All primary antibodies were applied for 2 hours at room temperature. After rinsing in PBS, secondary goat anti-mouse and goat anti-rabbit antibodies Alexa Fluor® 488 and Alexa Fluor® 568 (1∶300; Molecular Probes, Invitrogen) were applied for 1 h at room temperature in the dark. The slides were coverslipped with ProLong mounting media containing DAPI (Invitrogen). The staining specificity was tested by omission of primary antibodies. Immunostained preparations were examined and sections containing ventral horn at L4–L5 segmental levels were selected. Images were captured at 400x final magnification and 800–1600 µm rostral to the lesion site using a Nikon DXM1200 digital camera. The resulting images had a size of 1280×1024 pixels, which corresponded to 3.8 pixels per 1 µm tissue length. The area occupied by immunostained profiles was calculated using Image-Pro Plus software (Media Cybernetics, Inc., USA). Cell bodies of MAP2- and NF-labeled spinal motoneurons were manually outlined and excluded from the measurements.

### Western Blotting

Spinal cord segments were homogenized in buffer (pH 6.9) containing 5 mM EGTA, 100 mM PIPES, 5 mM MgCl_2_, 20% (v/v) glycerol, 0.5% (v/v) Triton X-100 and protease inhibitor cocktail (Sigma). The DC protein assay (Bio-Rad, Sweden) was performed to quantify sample protein levels. For each sample 10 µg of protein was denatured at 95°C and loaded onto a 10% SDS-polyacrylamide gel. The proteins were transferred to nitrocellulose membrane and then blots were blocked with 5% (w/v) non-fat milk in Tris buffered saline with Tween (TBS-T) and incubated with mouse anti-GFAP antibody (1∶500; NeoMarkers, USA), mouse anti-OX42 antibody (1∶200; Santa Cruz Biotechnology Inc, USA) or mouse anti-ED1 (1∶300; Abcam, UK) overnight at 4°C. Blots were then washed 6×5 min in TBS-T and incubated with mouse IgG HRP-conjugated secondary antibody (Cell Signaling Technology, USA) for 1h. The blots were then washed again 6×5 min and then exposed to ECL substrate (GE Healthcare, UK) for 1 min, and developed onto Kodak XPS films. The blots were re-probed with anti-actin (1∶5000; Millipore, Sweden) as a loading control. Films were scanned using an Epson Photoscanner and then analysed using Scion Image (Scion Corporation, Maryland, USA) which performs peak area integration to determine the area of each band in pixel units. The optical density of each protein was expressed as a ratio of the corresponding signal for β-actin.

### Image Processing

Preparations were photographed with a Nikon DXM1200 digital camera. The captured images were resized, grouped into a single canvas and labeled using Adobe Photoshop CS4 software. The contrast and brightness were adjusted to provide optimal clarity.

### Statistical Analysis

One-way analysis of variance (ANOVA) followed by a post hoc Newman-Keuls Multiple Comparison Test was used to determine statistical differences between the experimental groups (Prism®, GraphPad Software, Inc; San Diego, CA). Statistical significance was set as *p<0.05, **p<0.01, ***p<0.001.
